# Criticism upon the Use of “Volasen”

**Published:** 1899-12

**Authors:** 


### Domestic Correspondence.

### CRITICISM UPON THE USE OF "VOLASEN."

To the Editor:

Sir,—In the April number of the International Dental Journal, pages 259, 260, appears a part of the proceedings of the Northeastern Dental Association, wherein certain statements are made by Dr. G. Lennox Curtis, of New York, relative to the use of "volasen" by Dr. Howard Kelly, of Baltimore, in an operation for suspension of the bladder.

In the light of the enclosed letter from Dr. Kelly, it is fair to presume that Dr. Curtis has been misquoted in the report of the Association. Too often statements are made in society meetings, and thence published through the reports of the meetings, which, though taken by the older members of the profession *cum grano salis*, are liable to do the young practitioner much damage. On these grounds I have had occasion to investigate from time to time those statements which seemed to me to be destined to produce such a result with the younger members of the profession. The positive declaration of the article to which I call your attention, on account of the well-known dangers from idiosyncrasy incident to the use of cocaine, led me to make inquiry through a friend, who is a personal acquaintance of Dr. Kelly, as to the authenticity of the report. That there might be no mistake, I sent a copy of the Journal containing it, with the result as herein contained.

I assure you that the motive that actuates me in writing you is only to correct a mistake that possibly may have been made, in order that the younger members of the profession may not be misled by the positive statements made in the Journal and be induced to trust an antidote the components of which are unknown.

And again, believe me, I do not question the efficacy of the compound volasen, never having tried it, yet should untoward symptoms arise during its administration, even were they from some other cause, an explanation would be hard to make, or a legal investigation difficult to meet. Trusting the spirit with which these remarks are accompanied will be fully appreciated by all concerned, I remain

Respectfully yours,

B. S. Scott.

Tacoma Wash., July 19, 1899.

(The following is a copy of Dr. Kelly's letter.—Ed.)

"1406 Eutaw Place.

"Dear Doctor,—I operated on a patient last October, using cocaine alone for an anæsthetic.

"A warm personal friend of patient—Dr. Curtis, of New York—sent me a preparation, which he begged me to give her, at the same time; until I saw this note you send me I had forgotten its name. The dose was small, and I accepted his assurance it was not dangerous, and gave it.

"I do not know that it had the slightest effect, and have never used it since; and since seeing the article you send me never would use it again. It was used in a purely complaisant way, and I had no reason to attribute any effect to it whatever.

"Please make this statement very emphatically if my name is used any further.

"Truthfully yours,

(Signed) "Howard A. Kelly."

